# Genetic associations for keratoconus: a systematic review and meta-analysis

**DOI:** 10.1038/s41598-017-04393-2

**Published:** 2017-07-04

**Authors:** Shi Song Rong, Sarah Tsz Ue Ma, Xin Ting Yu, Li Ma, Wai Kit Chu, Tommy Chung Yan Chan, Yu Meng Wang, Alvin L. Young, Chi Pui Pang, Vishal Jhanji, Li Jia Chen

**Affiliations:** 1 Department of Ophthalmology and Visual Sciences, The Chinese University of Hong Kong, Hong Kong, China; 2 Department of Ophthalmology and Visual Sciences, Prince of Wales Hospital, Hong Kong, China; 3 Faculty of Medicine, The Chinese University of Hong Kong, Hong Kong, China; 4 Hong Kong Eye Hospital, Kowloon Hong Kong, China; 5 Department of Ophthalmology, Harvard Medical School, Massachusetts Eye and Ear, Boston, Massachusetts USA; 6 Department of Gastroenterology, Harvard Medical School, Beth Israel Deaconess Medical Center, Boston, Massachusetts USA; 7 UPMC Eye Centre, University of Pittsburgh School of Medicine, Pittsburgh, Pennsylvania USA

## Abstract

Genetic associations for keratoconus could be useful for understanding disease pathogenesis and discovering biomarkers for early detection of the disease. We conducted a systematic review and meta-analysis to summarize all reported genetic associations for the disease. We searched in the MEDLINE, Embase, Web of Science, and HuGENET databases for genetic studies of keratoconus published from 1950 to June 2016. The summary odds ratio and 95% confidence intervals of all polymorphisms were estimated using the random-effect model. Among 639 reports that were retrieved, 24 fulfilled required criteria as eligible studies for meta-analysis, involving a total of 53 polymorphisms in 28 genes/loci. Results of our meta-analysis lead to the prioritization of 8 single-nucleotide polymorphisms (SNPs) in 6 genes/loci for keratoconus in Whites. Of them 5 genes/loci were originally detected in genome-wide association studies, including *FOXO1* (rs2721051, P = 5.6 × 10^−11^), *RXRA-COL5A1* (rs1536482, P = 2.5 × 10^−9^), *FNDC3B* (rs4894535, P = 1.4 × 10^−8^), *IMMP2L* (rs757219, P = 6.1 × 10^−7^; rs214884, P = 2.3 × 10^−5^), and *BANP-ZNF469* (rs9938149, P = 1.3 × 10^−5^). The gene *COL4A4* (rs2229813, P = 1.3 × 10^−12^; rs2228557, P = 4.5 × 10^−7^) was identified in previous candidate gene studies. We also found SNPs in 10 genes/loci that had a summary P value < 0.05. Sensitivity analysis indicated that the results were robust. Replication studies and understanding the roles of these genes in keratoconus are warranted.

## Introduction

Keratoconus is a noninflammatory degenerative disorder that results in bulging and distortion of the corneal surface, leading to irregular astigmatism and progressive myopia. In advanced cases, corneal scarring and even corneal blindness can occur. Keratoconus has an incidence of approximately 1 in 2,000 individuals with a prevalence varying from 8.8 to 2300 per 100,000^1, 2^. It is a leading indication for corneal transplantation in many countries, especially in Australia, Middle East and Africa^3^. Management of keratoconus varies from conservative visual correction by spectacles or contact lenses for mild disease, to surgical interventions such as collagen cross-linking, intracorneal rings and keratoplasty for advanced disease. The onset of keratoconus is insidious and the progression is irreversible. Therefore, early diagnosis of keratoconus and its progression is needed. However, the variable risk of keratoconus progression poses a challenge to the personalized management for patients^4^. Knowing the risk factors for keratoconus would thus be helpful for early detection and monitoring the progression of the disease.

Keratoconus is a multifactorial disease resulting from the interaction of environmental, behavioural and genetic factors. Major environmental and behavioural factors include contact lens wear^5^ and chronic eye rubbing^6^. The genetic aetiology is evidenced by the bilaterality, familial aggregation^7–9^, monozygotic twins concordant of the disease^10^, its association with other genetic diseases such as Down syndrome^11^ and Leber’s congenital amaurosis^12^, and the ethnic difference in the prevalence and incidences^13^. Genetic associations for keratoconus will provide insight into disease mechanisms and help identify biomarkers for early detection of keratoconus onset and monitoring its progression. Of note, about 14% of the patients with keratoconus have a family history^9^. So far, however, the difference in the genetic basis of familial and sporadic keratoconus is unclear. Since the family history does not affect disease severity, the pooling of all cases in genetic studies is deemed reasonable^14^.

So far, 6 chromosomal loci have been identified for isolated keratoconus by linkage analysis, namely *2p24* ^15^, *3p14-q13* ^16^, *5q14.3-q21.1* ^2^, *13q32* ^18^, *16q22.3-q23.1* ^19^, and *20q12* ^20^. However, no disease-causing mutation has been identified from these loci. Besides, genome-wide association studies (GWAS) and candidate gene association studies have reported over 150 polymorphisms in more than 60 genes/loci for keratoconus. Among them, 7 genes/loci were identified by GWAS, including the *HGF* ^21^, *LOX* ^22^, *FOXO1* and *FNDC3B* genes^23^, and the *3p26*, *2q21.3* and *19q13.3* loci^24^. However, most of these associations were inconsistent across different study cohorts, making the roles of the genes/loci inconclusive.

In this study, we conducted a systematic review and meta-analysis to summarize the genetic association evidence for all variants in genes that were previously reported for keratoconus, and evaluated potential trans-ethnic heterogeneities. We first presented the association results from selected original studies/cohorts in the forest plots and then provided a prioritized list of studies and genes variants for further analysis. For SNPs that have been meta-analyzed in prior studies, our study provides an update of the summary association results by including new studies.

## Results

### Selection of studies

**Figure 1:**
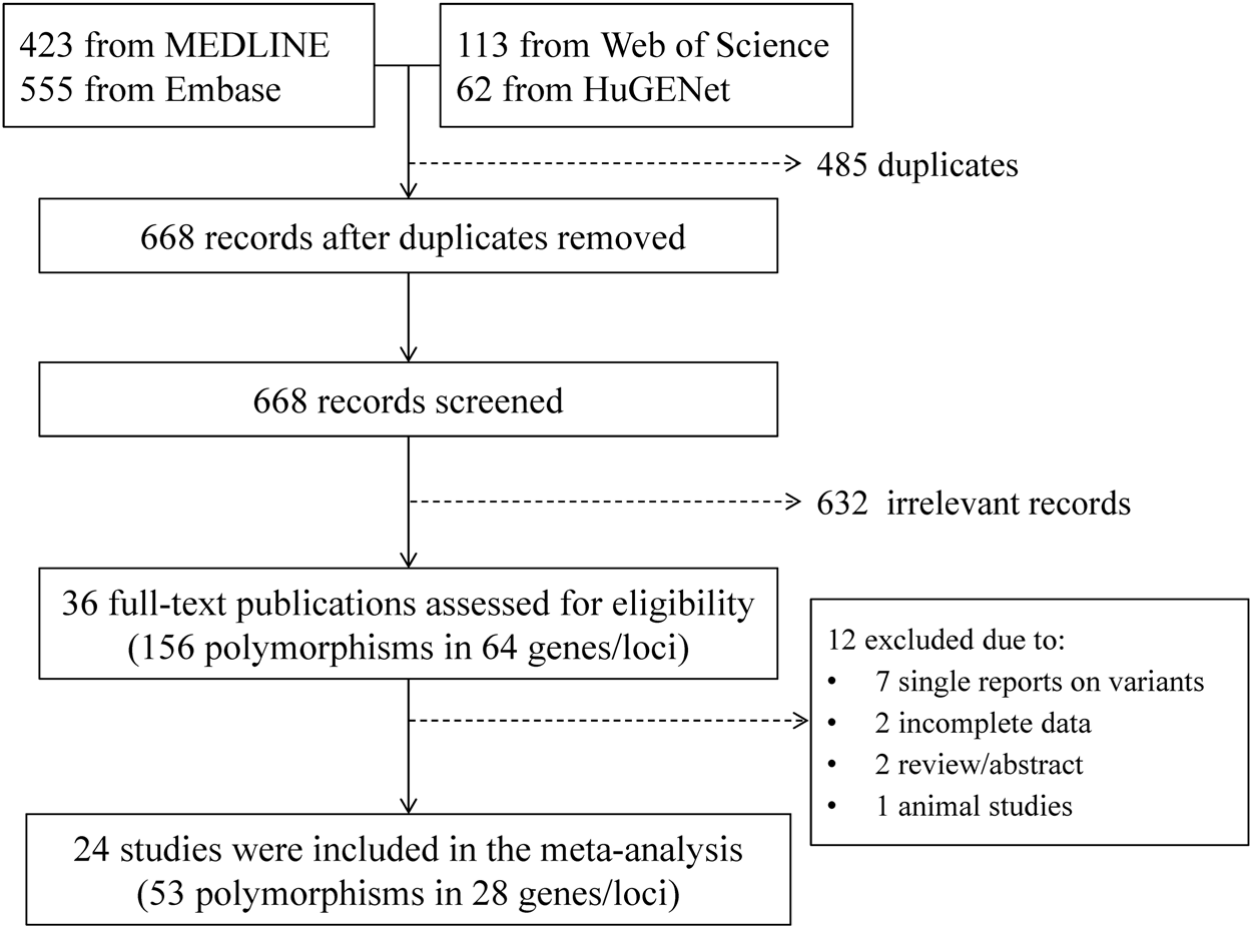
Flow diagram of study selection process.

We retrieved a total of 978 records published between 1950 and 1 June 2016 from MEDLINE, Embase, Web of Science, and HuGENET for review. After removing 339 duplicated records we evaluated 639 citations and selected 36 articles for full-text assessment. Among them, 2 were reviews^25, 26^ and 32 were molecular genetic studies, including 2 GWAS^23, 24^ and 30 candidate gene association studies. A total of 64 genes/loci and 156 variants have been identified from the full-text review (Supplementary Table 1; Fig. 1). In the meta-analysis, we excluded 12 of the 36 articles because 7 of them were about gene variants that were not tested in additional independent studies^27–33^, 2 reported insufficient genotype data for meta-analysis^34, 35^, 2 were reviews^25, 26^, and 1 was an animal study^36^. We did not receive genotype data after contacting some of the authors^34, 35^. Finally, 24 studies were included for meta-analysis, involving a total of 53 SNPs in 28 genes/loci (Fig. 1)^21–24, 32, 37–54^. Among these 24 studies, 20 were candidate gene studies conducted in different populations, including Whites^39, 41, 43, 44, 47, 49–52, 55^, Arabic^37, 38, 40^, Chinese^45, 56^, Korean^53, 54^, Japanese^48^, Indian^46^, and Turkish^42^. The total sample sizes from these candidate gene studies were 3,037 patients with keratoconus and 9,928 controls. The 2 GWAS included 2,333 keratoconus patients and 16,655 controls of Caucasian origin (Table 1)^23, 24^.

**Table 1:** Characteristics of eligible studies for the meta-analysis.

No.	First author (year)	Country	Ethnicity	Study design	Age		Sex (% Female)		Sample size		Gene and locus	Test for HWE
					Case	Control	Case	Control	Case	Control		
1	Abu-Amero, K. K.^40^	Saudi Arabia	Arabic	CG	28 ± 7	n.r.	0.54	n.r.	108	300	*BANP-ZNF469*, and 6 loci	In HWE
2	Dudakova, L.^39^	Czech	Whites	CG	37 ± 13	40 ± 14	0.35	0.41	165	193	*HGF* and *LOX*	In HWE
3	Hao, X. D.^56^	China	Chinese	CG	21 ± 6	27 ± 11	0.14	0.24	210	191	*HGF*, *LOX*, and 6 loci	In HWE
4	Hasanian-Langroudi, F.^38^	Iran	Arabic	CG	30 ± 13	30 ± 16	0.50	0.56	112	150	*LOX*	In HWE
5	Saravani, R.^37^	Iran	Arabic	CG	30 ± 13	30 ± 16	0.50	0.56	112	150	*COL4A4*	In HWE
6	Kokolakis, N. S.^43^	Greece	Whites	CG	33 ± 14	43 ± 16	0.38	0.44	45	78	*COL4A3* and *COL4A4*	In HWE
7	Karolak, J. A.^44^	Poland	Whites	CG	22–67	13–83	0.33	0.52	42	50	*VSX1*	n.r.
8	Sahebjada, S.^41^	Australia	Whites	CG	38 ± 16	53 ± 15	0.41	0.61	157	673	*HGF*	In HWE
9	Palamar, M.^42^	Turkey	Turkish	CG	25 ± 5	34 ± 12	0.54	0.51	121	121	*IL1B* & *IL1RN*	In HWE
10	Bae, H. A.^49^*	Australia	Whites	CG	43 ± 15	70 ± 10	0.45	0.43	524	2,761	12p13.3 and 11 loci	In HWE
11	Li, X.^55^	USA-1	Whites	C	44 ± 13	72 ± 5	0.45	0.61	222	3,324	*COL5A1*	n.r.
		USA-2	Whites	CG	43 ± 16	45 ± 14	0.32	0.48	304	518	*COL5A1*	n.r.
12	Sahebjada, S.^47^	Australia	Whites	CG	38 ± 16	53 ± 15	0.41	0.61	157	673	*BANP*-*ZNF469* and 4 loci	In HWE
13	Mikami, T.^48^	Japan	Japanese	CG	34 ± 10	33 ± 10	0.24	0.25	169	390	*IL1A* and *IL1B*	In HWE
14	Verma, A.^46^	India	Indian	CG	23 ± 6	25 ± 9	0.41	0.75	117	108	*VSX1*	n.r.
15	Lu, Y.^23^	Australia	Whites	GWAS	n.r.	n.r.	n.r.	n.r.	652	2,761	*BANP*-*ZNF469* and 4 loci	n.r.
		USA	Whites	CG	n.r.	n.r.	n.r.	n.r.	222	3,324	*BANP*-*ZNF469* and 4 loci	n.r.
16	Wang, Y.^45^	China	Chinese	CG	21 ± 6	22 ± 5	0.36	0.53	97	101	*COL4A3* and 4 loci	In HWE
17	Bykhovskaya, Y.^22^	USA-1	Whites	CG	44 ± 13	72 ± 5	0.45	0.61	222	3,324	*LOX*	n.r.
		USA-2	Whites	CG	43 ± 16	45 ± 14	0.32	0.48	304	518	*LOX*	n.r.
18	Li, X.^24^	USA-1	Whites	GWAS	44 ± 13	72 ± 5	0.45	0.61	222	3,324	12p13.3 and 11 loci	In HWE
		USA-2	Whites	CG	43 ± 16	45 ± 14	0.32	0.48	304	518	12p13.3 and 11 loci	In HWE
19	Burdon, K. P.^21^	Australia	Whites	CG	48 ± 16	77 ± 9	0.53	0.29	97	216	*HGF*	n.r.
		Australia	Whites	CG	43 ± 15	73 ± 11	0.39	0.10	96	72	*HGF*	n.r.
		Australia	Whites	CG	41 ± 15	72 ± 9	0.39	0.50	215	112	*HGF*	n.r.
		USA-1	Whites	CG	44 ± 13	72 ± 5	0.45	0.61	222	3,324	*HGF*	n.r.
		USA-2	Whites	CG	43 ± 16	45 ± 14	0.32	0.48	304	518	*HGF*	n.r.
20	Stabuc-Silih, M.^50^	Slovenia	Whites	CG	39 ± 10	n.r.	0.38	n.r.	113	100	*COL4A3* and *COL4A4*	n.r.
21	Stabuc-Silih, M.^51^	Slovenia	Whites	CG	39 ± 10	n.r.	0.38	n.r.	113	100	*VSX1*	n.r.
22	Stabuc-Silih, M.^52^	Slovenia	Whites	CG	39 ± 8	37 ± 10	0.38	0.36	104	157	*COL4A3* and *COL4A4*	In HWE
23	Kim, S. H.^54^	Korea	Korean	CG	18–33	n.r.	n.r.	n.r.	100	100	*IL1A* and 2 loci	In HWE
24	Mok, J. W.^53^	Korea	Korean	CG	n.r.	n.r.	n.r.	n.r.	249	208	*VSX1*	In HWE

^*^A small number of forme fruste Keratoconus was not excluded.

*BANP-ZNF469* = BTG3 associated nuclear protein-zinc finger protein 469; *COL4A3* = collagen, type IV, alpha 3; *COL4A4* = collagen, type IV, alpha 4; *COL5A1* = collagen, type V, alpha 1; *HGF* = hepatocyte growth factor; *IL1A* = interleukin 1, alpha; *IL1B* = interleukin 1, beta; *IL1RN* = interleukin 1 receptor antagonist; *LOX *= lysyl oxidase; *VSX1* = visual system homeobox 1.

CG = candidate gene association study; GWAS = genome-wide association study; KCN = keratoconus; HWE = Hardy Weinberg equilibrium; PCs = principle components; n.r. = not reported.

### Genes reported in keratoconus GWAS

**Figure 2:**
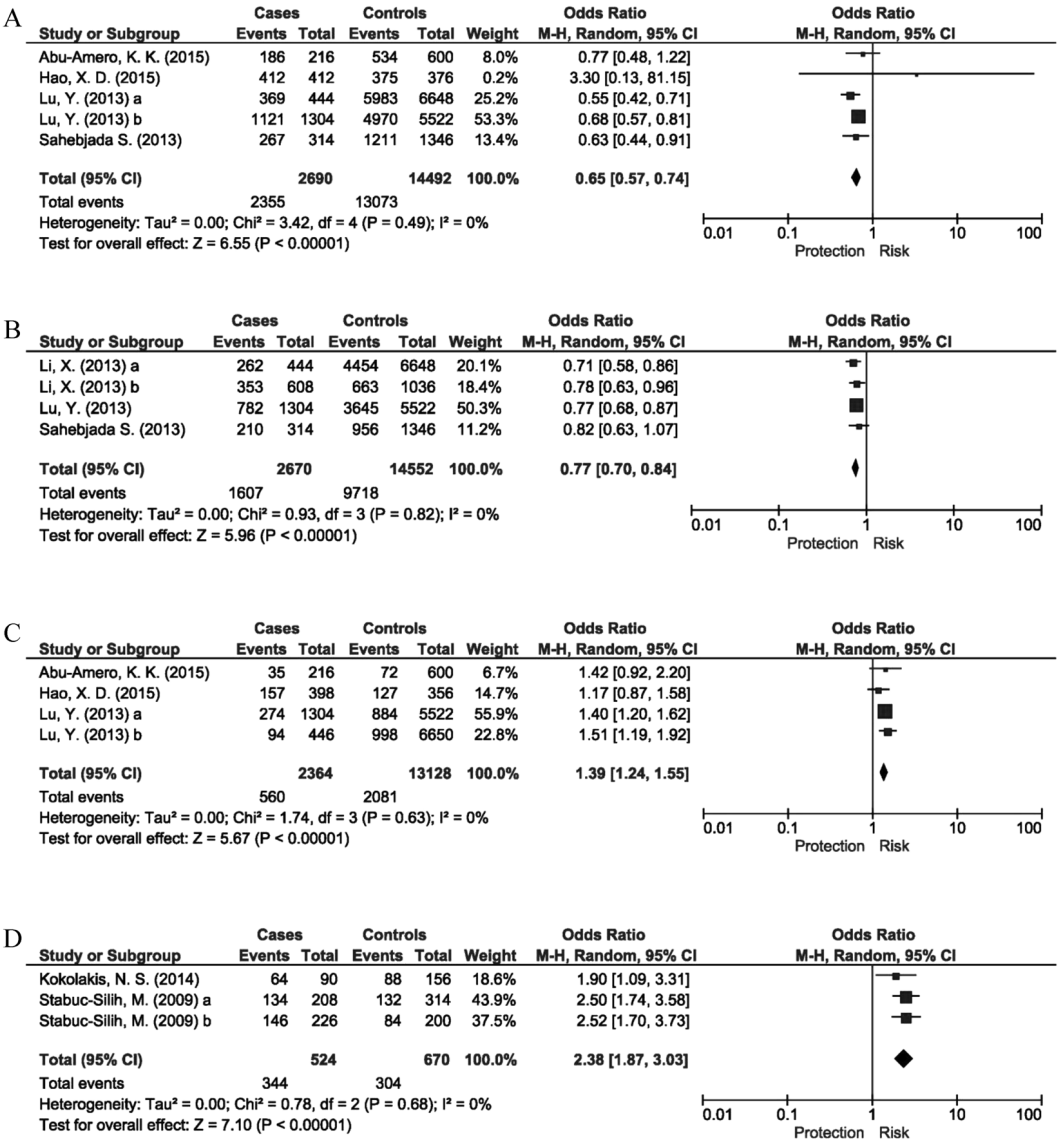
Meta-analysis of the 5 SNPs in 4 genes/loci showed genome-wide significance. Of the 4 genes/loci, 3 were detected in genome-wide association studies, including (**A**) *FOXO1* (rs2721051, P = 5.6 × 10^−11^, I^2^ = 0), (**B**) *RXRA-COL5A1* (rs1536482, P = 2.5 × 10^−9^, I^2^ = 0) and (**C**) *FNDC3B* (rs4894535, P = 1.4 × 10^−8^, I^2^ = 0). The (**D**) *COL4A4* (rs2229813, P = 1.3 × 10^−12,^ I^2^ = 0) gene was identified by candidate gene analysis.

We first meta-analyzed the SNPs that were reported in the four keratoconus GWAS^23, 24^ and additional independent studies based on the GWAS^21, 22, 39–41, 47, 49, 55, 56^. A total of 27 SNPs in 22 genes/loci were involved. Among them, 16 SNPs in 14 genes/loci showed a summary P value < 0.05 (Table 2). Of note, 3 SNPs in 3 respective genes/loci reached genome-wide significance, including *FOXO1* rs2721051 (P = 5.6 × 10^−11^, I^2^ = 0), *RXRA-COL5A1* rs1536482 (P = 2.5 × 10^−9^, I^2^ = 0), and *FNDC3B* rs4894535 (P = 1.4 × 10^−8^, I^2^ = 0) (Table 2 and Fig. 2). The P values for the remaining 13 significantly-associated SNPs ranged from 6.1 × 10^−7^ (*IMMP2L* rs757219) to 0.035 (*19p12* rs8111998) (Table 2).

**Table 2:** Allelic associations of gene variations with keratoconus using cohorts from both GWAS and subsequent replication studies.

No.	Gene/locus	SNP	No. of cohorts	Ethnicity	Associated allele vs. Reference allele	Pooled sample size		Outcome*		Heterogeneity		Egger’s test (P)
						Case	Control	P	OR (95% CI)	P (Q)	I^2^ (%)	
1	*FOXO1*	rs2721051	5	Multiple ancestries^†^	C vs. T	1,345	7,246	5.6 × 10^−11^	0.65 (0.57–0.74)	0.491	0	0.35
2	*RXRA-COL5A1*	rs1536482	4	Whites	G vs. A	1333	7276	2.5 × 10^−9^	0.77 (0.70–0.84)	0.819	0	0.89
3	*FNDC3B*	rs4894535	4	Multiple ancestries^†^	T vs. C	1,182	6,563	1.4 × 10^−8^	1.39 (1.24–1.55)	0.628	0	0.76
4	*IMMP2L*	rs757219	3	Whites	C vs. T	1,052	6,604	6.1 × 10^−7^	1.45 (1.25–1.67)	0.266	26	0.61
		rs214884	3	Whites	G vs. A	1,051	6,603	2.3 × 10^−5^	1.56 (1.27–1.91)	0.157	46	0.89
5	*BANP-ZNF469*	rs9938149	5	Multiple ancestries^†^	C vs. A	1,346	7,248	1.3 × 10^−5^	0.79 (0.70–0.88)	0.422	12	0.77
6	*KCND3*	rs4839200	2	Whites	A vs. G	745	6,084	3.9 × 10^−4^	1.63 (1.25–2.14)	0.068	70	n.a.
7	*RAB3GAP1*	rs4954218	3	Whites	G vs. T	1049	6604	8.2 × 10^−4^	0.64 (0.50–0.83)	0.021	75	0.19
8	*UBXD2*	rs6430585	3	Whites	A vs. C	1049	6604	1.1 × 10^−3^	1.36 (1.13–1.64)	0.065	63	0.62
9	*13q33.3*	rs1328089	2	Whites	C vs. T	747	6,086	1.7 × 10^−3^	1.38 (1.13–1.68)	0.109	61	n.a.
		rs1328083	3	Whites	G vs. T	1,050	6,604	3.0 × 10^−2^	1.38 (1.03–1.84)	0.008	82	0.88
10	*MPDZ-NFIB*	rs1324183	5	Multiple ancestries^†^	C vs. A	1,349	7,250	5.5 × 10^−3^	0.76 (0.63–0.92)	0.034	67	0.75
11	*COL5A1*	rs7044529	6	Multiple ancestries^†^	C vs. T	1,652	7,766	7.0 × 10^−3^	0.84 (0.74–0.95)	0.432	18	0.051
12	*LOX*	rs10519694	3	Whites	T vs. C	692	6,599	0.018	0.76 (0.61–0.95)	0.138	50	0.74
		rs2956540	4	Multiple ancestries^†^	G vs. C	901	6,788	0.28	0.83 (0.59–1.16)	<0.001	87	0.35
13	*HGF*	rs3735520	6	Multiple ancestries^†^	T vs. C	1,311	4,545	0.027	1.25 (1.03–1.51)	0.002	72	0.60
		rs1014091	2	Whites	A vs. G	362	480	0.41	0.70 (0.30–1.64)	0.005	87	n.a.
		rs2286194	2	Whites	A vs. T	354	960	0.70	0.85 (0.39–1.89)	0.001	91	n.a.
14	*19p12*	rs8111998	3	Whites	T vs. C	1,049	6,603	0.035	1.48 (1.03–2.13)	0.018	75	0.74
15	*PPP3CA*	rs2659546	3	Whites	A vs. G	1,050	6,602	0.06	1.46 (0.99–2.15)	0.014	75	0.77
16	*3q26.2*	rs6792542	3	Whites	C vs. A	1,051	6,603	0.15	1.22 (0.93–1.61)	0.001	84	0.48
17	*BHLHB2*	rs6442925	3	Whites	T vs. C	1,050	6,603	0.21	1.28 (0.87–1.88)	<0.001	89	0.93
18	*KIF26B*	rs12407427	2	Whites	T vs. C	747	6,085	0.28	1.34 (0.79–2.30)	0.001	92	n.a.
19	*BIRC8*	rs1428642	3	Whites	A vs. G	1,050	6,602	0.29	0.84 (0.61–1.16)	<0.001	90	0.45
20	*LRRN1*	rs3749350	3	Whites	T vs. G	1,052	6,603	0.32	1.24 (0.81–1.88)	<0.001	89	0.64
21	*12p13.3*	rs1978238	2	Whites	C vs. A	746	6,086	0.36	0.81 (0.52–1.27)	<0.001	92	n.a.
22	*COL4A3*	rs7606754	2	Multiple ancestries^†^	A vs. G	760	3,061	0.42	1.10 (0.87–1.38)	0.155	50	n.a.

^*^A random-effects model was used.

^†^Multiple ancestries included 2 or more ethnic groups from Whites and Asian (Arabic, Chinese, Korean, Japanese, or Indian).

CI = confidence interval; OR = odds ration; SNP = single nucleotide polymorphism; n.a. = not applicable; No. = number.

We then performed meta-analysis only using the candidate gene studies, including those based on the GWAS findings or other hypotheses. One SNP from GWAS was significantly associated with keratoconus, i.e., *RXRA-COL5A1* rs1536482 (P = 1.5 × 10^−5^, I^2^ = 0), while *FOXO1* rs2721051 (P = 9.4 × 10^−3^, I^2^ = 0), *BANP-ZNF469* rs9938149 (P = 0.017, I^2^ = 27%), *COL4A4* (rs2228557, P = 0.020, I^2^ = 70%) and *COL4A3* (c.2685 A > C, P = 0.032, I^2^ = 0) were nominally significant (Table 3). One SNP, *FNDC3B* rs4894535, reached a genome-wide significance in the overall population but did not show a significant association in the pooled Chinese and Arabic samples (P = 0.078, I^2^ = 0; Table 3). The other 4 genes/loci that have been reported in GWAS (i.e., *MPDZ-NFIB*, *COL5A1*, *LOX* and *HGF*) were also insignificant (P > 0.050; Table 3).

**Table 3:** Allelic associations of gene variations with keratoconus based on purely candidate gene studies.

No.	Gene/locus	SNP	No. of cohorts	Ethnicity	Associated allele vs. Reference allele	Pooled sample size		Outcome*		Heterogeneity		Egger’s test (P)
						Case	Control	P	OR (95% CI)	P (Q)	I^2^ (%)	
1	*RXRA-COL5A1*	rs1536482	3	Whites	G vs. A	681	4,515	1.5 × 10^−5^	0.76 (0.67–0.86)	0.64	0	0.44
2	*FOXO1*	rs2721051	3	Multiple ancestries^†^	C vs. T	471	1,162	9.4 × 10^−3^	0.69 (0.52–0.91)	0.51	0	0.28
3	*BANP-ZNF469*	rs9938149	3	Multiple ancestries^†^	C vs. A	472	1,164	0.017	0.75 (0.59–0.95)	0.31	27	0.40
4	*COL4A4*	rs2228557	4	Multiple ancestries^†^	T vs. C	359	437	0.020	0.63 (0.43–0.93)	0.021	70	0.41
		rs2229813	5	Multiple ancestries^†^	G vs. A	471	588	0.18	1.46 (0.84–2.55)	1.2 × 10^−8^	90	0.67
		rs1800516	2	Whites	C vs. G	217	257	0.62	0.84 (0.42–1.66)	0.90	0	n.a.
		rs2228555	3	Multiple ancestries^†^	G vs. A	329	407	0.74	1.04 (0.84–1.28)	0.96	0	0.95
		rs2229814	3	Multiple ancestries^†^	T vs. C	315	358	0.78	1.03 (0.83–1.28)	0.86	0	0.72
		rs56247709	2	Whites	A vs. T	217	257	1.00	1.00 (0.51–1.95)	0.99	0	n.a.
5	*COL4A3*	c.2685 A > C	2	Whites	C vs. A	217	258	0.032	1.36 (1.03–1.79)	0.98	0	n.a.
		rs55703767	4	Multiple ancestries^†^	T vs. G	360	436	0.14	0.29 (0.06–1.48)	5.1 × 10^−16^	96	0.18
		rs34019152	3	Multiple ancestries^†^	A vs. G	314	357	0.27	0.80 (0.53–1.19)	0.95	0	0.74
		rs28381984	3	Multiple ancestries^†^	T vs. C	314	359	0.27	0.89 (0.71–1.10)	0.92	0	0.74
		rs11677877	3	Multiple ancestries^†^	G vs. A	315	357	0.58	0.90 (0.62–1.30)	0.77	0	0.49
		rs13424243	3	Multiple ancestries^†^	C vs. G	314	358	0.67	0.89 (0.51–1.54)	0.51	0	0.30
		rs6436669	3	Multiple ancestries^†^	G vs. A	314	359	0.92	1.02 (0.75–1.38)	0.94	0	0.73
		rs10178458	3	Multiple ancestries^†^	T vs. C	313	358	0.97	0.99 (0.73–1.34)	0.93	0	0.70
6	*FNDC3B*	rs4894535	2	Chinese and Arabic	T vs. C	307	477	0.078	1.25 (0.98–1.60)	0.48	0	n.a.
7	*VSX1*	rs12480307	3	Multiple ancestries^†^	G vs. A	256	259	0.14	1.34 (0.91–1.98)	0.30	0	0.23
		rs8123716	2	Multiple ancestries^†^	A vs. C	139	152	0.27	1.58 (0.70–3.57)	0.34	0	n.a.
		rs74315433	2	Multiple ancestries^†^	T vs. G	139	151	0.48	1.76 (0.36–8.55)	0.26	23	n.a.
		rs56157240	2	Chinese and Indian	T vs. A	214	209	0.53	1.80 (0.28–11.40)	0.075	69	n.a.
		rs6138482	5	Multiple ancestries^†^	A vs. G	614	555	0.70	1.05 (0.83–1.32)	0.20	36	0.060
8	*COL5A1*	rs7044529	5	Multiple ancestries^†^	C vs. T	1,001	5,005	0.17	0.90 (0.78–1.04)	0.80	0	0.34
9	*MPDZ-NFIB*	rs1324183	3	Multiple ancestries^†^	C vs. A	474	1,164	0.18	0.75 (0.5–1.14)	7.3 × 10^−3^	81	0.051
10	*IL1A*	rs2071376	3	Korean, Chinese, and Japanese	A vs. C	366	590	0.33	1.15 (0.87–1.52)	0.16	43	0.89
11	*IL1B*	rs16944	4	Multiple ancestries^†^	T vs. C	487	711	0.52	0.91 (0.69–1.21)	0.047	63	0.51
		rs1143627	3	Korean, Chinese, and Japanese	C vs. T	366	591	0.53	0.87 (0.58–1.33)	0.017	77	0.58
12	*IL1RN*	rs2234663	2	Multiple ancestries^†^	1 vs. Non-1^‡^	221	221	0.93	0.98 (0.69–1.4)	0.58	0	n.a.
		rs2234663	2	Multiple ancestries^†^	2 vs. Non-2^‡^	221	221	0.65	1.16 (0.61–2.18)	0.15	51	n.a.
		rs2234663	2	Multiple ancestries^†^	3 vs. Non-3^‡^	221	221	0.84	0.92 (0.4–2.13)	0.47	0	n.a.
		rs2234663	2	Multiple ancestries^†^	4 vs. Non-4^‡^	221	221	0.53	0.62 (0.14–2.75)	0.47	0	n.a.
13	*HGF*	rs3735520	2	Multiple ancestries^†^	T vs. C	375	382	0.57	1.14 (0.72–1.81)	0.025	80	n.a.
14	*LOX*	rs2288393	2	Multiple ancestries^†^	C vs. G	276	342	0.72	1.11 (0.63–1.95)	0.057	72	n.a.
		rs1800449	2	Multiple ancestries^†^	C vs. T	277	343	0.84	0.92 (0.42–2.04)	4.0 × 10^−3^	88	n.a.
		rs2956540	2	Multiple ancestries^†^	G vs. C	375	383	0.97	0.99 (0.47–2.10)	6.8 × 10^−4^	91	n.a.

^*^A random-effects model was used.

^†^Multiple ancestries included 2 or more ethnic groups from Whites and Asian (Arabic, Chinese, Korean, Japanese, or Indian).

^‡^ *IL1RN* rs2234663 were designated as *IL1RN*∗1 [4 repeats, 410 base pairs (bp)], *IL1RN*∗2 (2 repeats, 240 bp), *IL1RN*∗3 (5 repeats, 500 bp), *IL1RN*∗4 (3 repeats, 325 bp), and *IL1RN*∗5 (6 repeats, 595 bp).

CI = confidence interval; OR = odds ration; SNP = single nucleotide polymorphism; n.a. = not applicable; No. = number.

### Stratification analysis

To reduce the potential impact of trans-ethnical heterogeneity to the overall genetic association, we grouped the study cohorts into Whites and others (including Chinese, Korean, Japanese, Indian and Arabic). The 5 SNPs that were identified from GWAS showed a robust or nominal significance in Whites: *FOXO1* rs2721051 (P = 1.5 × 10^−9^, I^2^ = 11%), *MPDZ-NFIB* rs1324183 (P = 1.8 × 10^−4^, I^2^ = 49%), *BANP-ZNF469* rs9938149 (P = 2.6 × 10^−4^, I^2^ = 42%), *COL5A1* rs7044529 (P = 9.9 × 10^−4^, I^2^ = 12%) and *HGF* rs3735520 (P = 3.6 × 10^−3^, I^2^ = 66%; Supplementary Table 2). Moreover, 2 SNPs in the *COL4A4* gene identified by candidate gene studies were strongly associated with keratoconus in Whites, namely rs2229813 (P = 1.3 × 10^−12^, odds ratio (OR) = 2.38; I^2^ = 0) and rs2228557 (P = 4.5 × 10^−7^, OR = 0.54; I^2^ = 0) (Supplementary Table 2 and Fig. 2). In contrast, SNP rs2229813 showed a nominal association with keratoconus in combined Chinese and Arabic samples (P = 0.047, I^2^ = 16%). The odds ratio was notably toward an opposite direction (OR = 0.74; Supplementary Table 2). Moreover, most of aforementioned significant SNPs in Whites were not significant in the Chinese and Arabic samples, including *FOXO1* rs2721051 (P = 0.31; I^2^ = 0), *BANP-ZNF469* rs9938149 (P = 0.32; I^2^ = 0), *MPDZ-NFIB* rs1324183 (P = 0.63; I^2^ = 82%), and *COL5A1* rs7044529 (P = 0.95; I^2^ = 0) (Supplementary Table 2), indicating ethnic diversities.

In this study, we were not able to evaluate the potential difference in the genetic basis of familial and sporadic cases as the data from familial cases were limited.

### Assessment of potential biases and sensitivity analysis

For quality assessment every study was awarded a star for each of the items, i.e., case definition, ethnicity, and ascertainment of genotype (Supplementary Table 3) according to the Newcastle Ottawa Scale (NOS) system. All the 24 studies were awarded 5 or more stars out of a maximum of 8. Regarding Hardy-Weinberg Equilibrium (HWE), the control groups in 3 study cohorts showed deviation from HWE when tested for *FOXO1* (rs2721051)^56^, *COL4A3* (rs10178458 and rs55703767)^52^, *COL4A4* (rs2229813, rs2228555, and rs2229814)^52^, and *VSX1* (rs12480307)^45^. Therefore, in the sensitivity analysis we first excluded all the cohorts with HWE deviation and recalculated the summary ORs for the 7 SNPs in 4 genes. The associations were not altered (Supplementary Table 4). Furthermore, we omitted each study one at a time sequentially and recalculated the summary outcomes. The significance or insignificance of the summary outcomes was not altered in the sensitivity analysis (data not shown). We did not detect significant small study effects (e.g. publication bias) according to the shapes of funnel plots (Supplementary Figure 1) and the P values from the Egger’s tests, except for *COL4A3* (rs55703767), *LOX* (rs2956540) and *VSX1* (rs6138482) in the subgroup analysis by ethnicity (Supplementary Table 1).

## Discussion

In this study, we meta-analyzed a total of 53 SNPs in 28 genes/loci for their genetic associations with keratoconus. We identified 8 SNPs in 6 genes/loci that were associated with keratoconus, i.e., *FOXO1* rs2721051, *FNDC3B* rs4894535 and *BANP-ZNF469* rs9938149 for the overall combined cohorts, and *RXRA-COL5A1* rs1536482, *IMMP2L* rs757219 and rs214884, and *COL4A4* rs2229813 and rs2228557 for Whites. Also, we found nominally significant associations in another 10 genes/loci, including *KCND3*, *RAB3GAP1*, *UBXD2*, *MPDZ-NFIB*, *COL5A1*, *LOX*, *HGF*, *COL4A3*, *13q33.3*, and *19p12*. In contrast, SNPs in 10 genes/loci that were reportedly associated with keratoconus were insignificant in our meta-analysis, including *BHLHB2*, *BIRC8*, *IL1A*, *IL1B*, *KIF26B*, *LRRN1*, *PPP3CA*, *VSX1*, *12p13.3* and *3q26.2*.

Among the 6 significant genes/loci for keratoconus, 5 were originally identified by GWAS, including *FOXO1*, *FNDC3B, BANP-ZNF469*, *RXRA-COL5A1*, and *IMMP2L*. In our meta-analysis involving data from the GWAS and independent replication studies, 3 genes/loci (i.e., *FOXO1*, *FNDC3B, BANP-ZNF469*) showed consistent effects with low heterogeneity across different study cohorts. Three of them, *FOXO1* rs2721051, *FNDC3B* rs4894535 and *BANP-ZNF469* rs9938149, have been tested in both Whites and Asian populations. However, none of them showed a significant association in Chinese^32^ or Arabs^40^. Of note, *FOXO1* rs2721051 was rare in Chinese with a minor allele frequency of 0.1%^32^. The lack of significant association in Asians could be due to the small sample size. In this meta-analysis, we also identified a SNP rs2229813 in the *COL4A4* gene that showed a summary P value of genome-wide significant in Whites (P = 1.3 × 10^−12^; OR = 2.38). This gene was identified only in candidate gene studies^37, 43, 45, 50, 52^. Interestingly the summary P value in the pooled non-Caucasian samples was nominally significant (P = 0.047), but the OR was toward the opposite direction (OR = 0.74). This may suggest trans-ethnic diversities in the genetic components of keratoconus. In the *COL4A4* gene, another SNP rs2228557, which was proposed in candidate gene studies, showed a significant summary P value (P = 4.5 × 10^−7^) in Whites, suggesting *COL4A4* could be a genuine susceptibility gene for keratoconus in Whites. However, rs2228557 has only been tested in a Chinese population showing an insignificant association with an opposite OR (1.09)^45^. Therefore, whether *COL4A4* is a biomarker with differential effects on keratoconus among different ethnic groups has yet to be confirmed. Interestingly, these 2 SNPs (i.e., rs2229813 and rs2228557) have not been reported in the published GWAS papers. In GWAS, only SNPs with P values surpassing a certain threshold would have been subjected to replication. Therefore, it would be intriguing to check the *COL4A4* SNPs in the GWAS data and assess their association with keratoconus.

Although we were not able to evaluate the potential difference in the genetic basis of familial and sporadic cases, we found 2 familial cohorts being tested for different genes/loci^22, 24, 50, 51, 55^. In 3 studies^22, 24, 55^, the authors tested the associations of a few genes/loci (e.g. *LOX* and *COL5A1*) with keratoconus in a familial cohort using a generalized estimating equation accounting for familial correlations. Some of the significant SNPs identified in unrelated cases also showed significant association with keratoconus in the familial cohort. In another 2 studies^50, 51^, the authors reported a mutation, “627 + 23 G > A”, in *VSX1* that was segregated in cases in several families. However, the mutation did not show significant association with keratoconus in the analysis using all the cases^50^. The results from the 2 cohorts indicated that the genetic association profiles of sporadic and familial keratoconus could be different.

Results of the present meta-analysis have led to a list of genes and loci associated with keratoconus that can be considered for functional investigations. Further biological investigation on these genes may throw light on new disease mechanisms for keratoconus. For example, *FOXO1*, *RXRA* and *FNDC3B* are the 3 genes that showed genome-wide significant association with keratoconus. *FOXO1* is a member of the Forkhead Box (Fox) transcription factor family. Proteins from this family contain a conserved forkhead domain, which is a 110 amino acid DNA-binding domain. Fox proteins are known to be important regulators of the cellular oxidative stress^57^. For example, Fox proteins regulate the expressions of anti-oxidative enzymes such as superoxide dismutase and thioredoxin reductase^58, 59^. Moreover, reduced FOXO1 expression has been reported to induce apoptosis in human trabecular meshwork cells in response to oxidative stress^60^. It has been shown that increased oxidative damage to trabecular meshwork cells results in elevation of intraocular pressure and changing the anterior chamber angle, which would lead to corneal thinning^61^. We also found association of keratoconus with *IMMP2L*, a mitochondrial inner membrane protease. Mutation in *IMMP2L* also accumulates oxidative stress^62^. Therefore, FOXO1 and IMMP2L might regulate the oxidative stress in the anterior chamber, which affects the intraocular pressure and the corneal thickness. FOXO1 has also been linked to adipocyte differentiation^63^, which is affected by the gene *FNDC3B* ^64^. In this study, *FNDC3B* is another keratoconus associated gene. The link between adipogenesis and keratoconus is currently unclear. However, *FNDC3B* was associated with elevated intraocular pressure in a GWAS study^65^. Hence, FNDC3B may influence the intraocular pressure, the anterior chamber angle and the corneal thickness. Another keratoconus gene is *RXRA*, which encodes a nuclear retinoic acid receptor protein. There are two classes of nuclear retinoic acid receptors: RXR and RAR, which bind to each other to form RXR/RAR heterodimers^66^. Null mice of both *RXRA* and *RXRA/RAR* showed abnormal embryonic eye morphologies, including thickening of corneal stroma and absence of anterior chamber^66^. These results suggest a potential role of *RXRA* and retinoic acid signaling in the ocular development. However, the link among retinoic acid signalling, ocular development, and the abnormal corneal in keratoconus remains to be explored.

It is noteworthy that all of the identified SNPs in the 16 genes/loci are located in intronic, inter-genic, or in 3′- or 5′-untranslated regions. One SNP in *HGF*, rs3735520 (c.−1652C > T), was reported to modulate the severity of interstitial lung disease in patients with systemic sclerosis by altering the transcriptional efficiency of the *HGF* gene^67^. Whether they are in linkage disequilibrium with other coding variants in the relevant genes remained to be elucidated by sequencing analyses.

Although the mechanisms underlying the significant associations of the 16 identified genes/loci with keratoconus are largely unknown, it might be useful for understanding their pathogenic effects by referring to disease pathways identified for other conditions that share the same genes/loci. Eleven genes have been implicated in other diseases, including: *COL5A1* for Ehlers-Danlos syndrome^68^; *COL4A3* and *COL4A4* for Alport syndrome^69^; *HGF* for non-syndromic hearing loss^70^; *IMMP2L* for Gilles de la Tourette syndrome^71^; *KCND3* for spinocerebellar ataxia^72^; *LOX* for thoracic aortic aneurysms and dissections^73^; *MPDZ* for leber congenital amaurosis and retinitis pigmentosa^74^; *RAB3GAP1* for Warburg Micro syndrome and Martsolf syndrome^75^; and *ZNF469* for Brittle cornea syndrome^76^. The other 6 of the 16 identified genes, namely *FOXO1*, *RXRA, FNDC3B, BANP*, *UBXD2*, and *NFIB* of the *MPDZ-NFIB* locus, have not been directly linked to other human diseases.

In this study, we have identified and evaluated the genetic associations for keratoconus by conducting thorough assessments of the existing evidence. We have taken multiple measures to control for potential biases, including subgroup analysis, sensitivity analysis, and Egger’s test. However, this study has some limitations. First, our results could be more applicable to Whites, therefore most of the significant findings should be replicated in other populations with sufficient statistical power, such as the Asian populations. Second, the sample sizes in most of the candidate gene studies were small, especially in Asian populations. We observed lack of associations of almost all SNPs when summarizing the data from Asian cohorts. Therefore, larger cohorts are needed for further validation. Third, although we employed funnel plots and Egger’s tests to identify publication bias, there could still be remaining publication bias due to the reduced power when testing small number of studies in a meta-analysis. Moreover, the *COL4A4* variants might not reach the genome-wide significance in the reported GWAS. The non-availability of the data for these variants could be a potential source of publication bias.

In conclusion, we have prioritized 8 SNPs in 6 genes/loci as significant genetic markers for keratoconus in Whites, including *FOXO1* rs2721051, *RXRA-COL5A1* rs1536482, *FNDC3B* rs4894535, *IMMP2L* rs757219 and rs214884, and *BANP-ZNF469* rs9938149, and *COL4A4* rs2229813 and rs2228557. We also identified 10 genes/loci with suggestive evidence of association with keratoconus. This study has thus provided an up-to-date list of candidate genetic markers for further investigations of their biological roles in keratoconus. More studies are warranted to confirm the reported genetic associations in different populations.

## Methods

### Searching methods for identifying studies

We searched for relevant records in the MEDLINE, Embase, Web of Science, and HuGENET databases via the Ovid platform. We used the Boolean logic to connect the free terms and controlled vocabularies (i.e. Medical Subject Heading terms): (“polymorphism(s)” OR “mutation” OR “genotype(s)” OR “genetic(s)” OR “gene(s)” OR “allele(s)” OR “DNA”) AND (“keratoconus”) (Supplementary Table 5). We also manually scanned the reference lists of the potentially eligible research articles, reviews and meta-analyses from the initial screening to ensure inclusion of all relevant publications. We did not use language filter in the literature search. The last search was performed on June 1, 2016.

### Eligibility criteria

We set the following criteria for eligible studies for meta-analysis: (1) original case-control studies that evaluated the association of gene polymorphisms with keratoconus; (2) the study subjects were unrelated and recruited from explicitly defined populations; and (3) allele or genotype counts or frequencies in both case and control groups were reported or calculable; or odds ratio and 95% confidence intervals (CI) and/or standard error (SE) were reported. We excluded animal studies, case reports, reviews, abstracts, conference proceedings, and editorials.

### Study selection, data collection and risk of bias assessment

Two reviewers (S.S.R. and S.T.U.M.) independently screened all the titles and abstracts of identified studies. Disagreements were resolved via discussions with a senior investigator (L.J.C.). After identifying potentially eligible articles, the 4 reviewers (S.S.R., S.T.U.M., X.T.Y., and L.M.) extracted the data separately and cross-validated the data. Consensus was reached via thorough discussion among all the reviewers. In this study, we used ‘Whites’ to represent individuals/populations whose ancestral origins are in the continent of Europe. We designed a customized datasheet for data extraction, which included the first author, year of publication, country of study, ethnicity, definition of case and control, sample sizes in the case and control groups, genes/loci, polymorphisms, allelic and genotypic counts and frequencies, ORs and 95% CIs or SEs of the polymorphisms and corresponding genetic models, and results of the test for HWE in the control group. First, we extracted all the polymorphisms and genes/loci reported in the potentially eligible studies searchable by the end of our search date. For GWAS, we extracted all the variants that were shown to be tested in replication cohorts in the result section and supplementary tables^21–24^. For candidate gene study, we extracted all the reported variants. No significance threshold for the genetic association has been applied during the data extraction. We also checked for potentially duplicated cohorts among the studies via comparing research groups and description of study populations. In the studies that had reported 2 or more independent cohorts, we extracted the data of each cohort separately. Second, we selected those polymorphisms that could be meta-analyzed. Third, the missing allele/genotype counts were calculated using the allele frequencies and sample sizes, assuming no deviation from HWE unless reported otherwise^77^. If only the OR and 95% CI were reported, we estimated the SE following the methods described in our previous papers^77, 78^. We attempted to contact the authors for additional information if necessary. If the HWE result was not reported, we tested it using the extracted data in the control group by the Chi-square test. Moreover, we used the NOS system (accessed via http://www.ohri.ca/programs/clinical_epidemiology/oxford.asp) to evaluate the quality of the case-control studies (Supplementary Appendix 1)^79, 80^. We assigned one star to a study if it met one requirement in the NOS from 3 dimensions (i.e., selection, comparability and exposure). The maximum number of stars that a study could obtain was 8. A study of <5 stars in overall or earned no star in any one of the items (i.e., case definition, ethnicity, or ascertainment of genotype) was considered as of suboptimal quality and having high risk in introducing bias^81^.

### Data analysis

We conducted meta-analysis for the SNPs that had been reported in 2 or more study cohorts from at least 2 separated reports. The genetic association was assessed using the allelic (a vs. A) model, where “a” and “A” represented the associated and the reference alleles, respectively. We evaluated the inter-cohort heterogeneity using the *I* ^*2*^ ^82^. An *I* ^*2*^ value of lower than 25%, between 25% and 50%, and greater than 50% indicated low, moderate, and high heterogeneity, respectively. However, to obtain more conservative results we calculated the summary OR and 95% CI for each SNP only using the random-effect model, in which the weighted effect of a SNP was estimated by inverse variance (IV) and τ^2^ from the DerSimonian-Laird estimator^83^, regardless of the Q statistics and *I* ^*2*^. Of note, to assess the replication results of SNPs identified in the GWAS on keratoconus^23, 24^, we first combined the data from both the GWAS and replication studies, and then removed the data from the initial GWAS. Subgroup analysis by ethnicity was then conducted in Whites and Asian populations (i.e., populations of Asian ancestries including 2 or more ethnic groups from Arabic, Chinese, Korean, Japanese, or Indian populations). We adopted the funnel plots and Egger’s test to assess potential biases (e.g. publication bias)^84, 85^. A P value of <0.05 in the Egger’s test indicated statistically significant bias. We also conducted the sensitivity analysis to confirm the associations by sequentially omitting each of the included studies one at a time and recalculated the summary outcomes. We then omitted the studies that deviated from HWE (P_Chi-squre_ ≤ 0.05), or of suboptimal quality. A finding is more likely to be true when the result is stable in the sensitivity analysis.

Customized analytical scripts based on the metafor package in the R software (v3.0.0, http://cran.r-project.org/) were generated for the meta-analysis.

As a strategy to account for multiple testing, we used the Bonferroni corrected alpha as the cut-off value for confirming the genetic associations. To calculate the adjusted alpha value, we divided 0.05 by the number of SNPs tested (N = 53) and also by the maximum number of different tests a SNP could be done (N = 7). The adjusted significant threshold was therefore 1.35 × 10^−4^. The P values > 1.35 × 10^−4^ and ≤ 0.05 were considered nominally significant. We consider a P value < 5 × 10^−8^ as genome-wide significance.

## Electronic supplementary material

Supplementary information
