# Supplementary material for: Genetic associations for keratoconus: a systematic review and meta-analysis
Source: Sci Rep. 2017 Jul 4;7:4620. doi: 10.1038/s41598-017-04393-2 (PMC5496893; doi:10.1038/s41598-017-04393-2)
Supplement: Supplementary file 1 — Supplementary information [file 41598_2017_4393_MOESM1_ESM.pdf]

Genetic associations for keratoconus: a systematic review and meta-analysis

Shi Song Rong[1,5], Sarah Tsz Ue Ma[1], Xinting Yu[1,3], Li Ma[1], Wai Kit Chu[1], Tommy Chung Yan Chan[4], Yu Meng Wang[1], Alvin L. Young[1,2], Chi Pui Pang[1], Vishal Jhanji[1,2], Li Jia Chen[1,2]

[1] Department of Ophthalmology and Visual Sciences, The Chinese University of Hong Kong, Hong Kong, China;

[2] Department of Ophthalmology and Visual Sciences, Prince of Wales Hospital, Hong Kong, China;

[3] Faculty of Medicine, the Chinese University of Hong Kong, Hong Kong, China;

[4] Hong Kong Eye Hospital, Kowloon, Hong Kong, China;

[5] Current affiliation: Department of Ophthalmology, Harvard Medical School, Massachusetts Eye and Ear, Boston, Massachusetts, USA.

[6] Current affiliation: Department of Gastroenterology, Harvard Medical School, Beth Israel Deaconess Medical Center, Boston, Massachusetts, USA.

List of supplementary materials:

Supplementary Table 1. Summary of reported and meta-analysed genes/loci and polymorphisms for keratoconus

Supplementary Table 2. Allelic associations of gene variations with keratoconus in different populations

Supplementary Table 3. Quality assessment of case-control study using Newcastle - Ottawa scale (NOS)

Supplementary Table 4. Sensitivity analysis of allelic associations by excluding cohorts deviated from Hardy-Weinberg Equilibrium

Supplementary Table 5. Search strategies for MEDLINE and Embase databases on the Ovid platform

Supplementary Figure 1. Funnel plots of significant genetic associations for keratoconus

Supplementary Appendix 1. Modified Newcastle - Ottawa Quality Assessment Scale for case-control studies

Supplementary Table 1. Summary of reported and meta-analysed genes/loci and polymorphisms for keratoconus

	Offical Symbol	No. of SNPs reported	No. of SNPs meta-analysed
1	*VSX1*	19	5
2	*HGF*	15	3
3	*CAST*	14	0
4	*COL4A3*	11	9
5	*COL4A4*	7	6
6	*IL1B*	5	2
7	*IL1RN*	5	1
8	*LOX*	5	4
9	*COL5A1*	4	2
10	*IL1A*	3	1
11	*TF*	3	0
12	*TGFBI*	3	0
13	*13q33.3*	2	2
14	*CHSY1*	2	0
15	*FEN1*	2	0
16	*FGF9-SGCG*	2	0
17	*HS3ST3B1-PMP22*	2	0
18	*IMMP2L*	2	2
19	*MPDZ-NFIB*	2	1
20	*RAD51*	2	0
21	*SMAD3*	2	0
22	*XRCC1*	2	0
23	*12p13.3*	1	1
24	*19p12*	1	1
25	*3q26.2*	1	1
26	*ADAMTS6*	1	0
27	*ARHGAP20-POU2AF1*	1	0
28	*ARID5B*	1	0
29	*BANP-ZNF469*	1	1
30	*BHLHE40*	1	1
31	*BIRC8*	1	1
32	*C7orf42*	1	0
33	*COL8A2*	1	0
34	*DOCK9*	1	0
35	*FAM46A-IBTK*	1	0
36	*FAS*	1	0
37	*FASLG*	1	0
38	*FNDC3B*	1	1
39	*FOXO1*	1	1
40	*GLT8D2*	1	0
41	*hsa-mir-568*	1	0
42	*KCND3*	1	1
43	*KIF26B*	1	1
44	*LCN12-PTGDS*	1	0
45	*LPAR1*	1	0
46	*LRRK1*	1	0
47	*LRRN1*	1	1
48	*MIR184*	1	0
49	*MUTYH*	1	0
50	*NEIL1*	1	0
51	*NR3C2*	1	0
52	*OGG1*	1	0
53	*PARP1*	1	0
54	*POLG*	1	0
55	*PPP3CA*	1	1
56	*PTGDS*	1	0
57	*RAB3GAP1*	1	1
58	*RXRA-COL5A1*	1	1
59	*SOD1*	1	0
60	*TBL1XR1-KCNMB2*	1	0
61	*TJP1*	1	0
62	*UBXN4*	1	1
63	*VKORC1L1*	1	0
64	*WNT10A*	1	0

No. = number; SNPs = single nucleotide polymorphism.

Supplementary Table 2. Allelic associations of gene variations with keratoconus in different populations

No.	Gene/locus	SNP	No. of cohorts	Ethnicity	Associated allele vs. Reference allele	Pooled sample size		Outcome*		Heterogeneity		Egger's test (P)
						Case	Control	P	OR (95% CI)	P (Q)	I^2 (%)	
**Based on both GWAS and replication studies**												
1	*FOXO1*	rs2721051	3	Whites	C vs. T	1,031	6,757	**1.5×10^{-9}**	0.63 (0.54 - 0.73)	0.40	11	0.56
2	*MPDZ-NFIB*	rs1324183	3	Whites	C vs. A	1,032	6,759	**1.8×10^{-4}**	0.72 (0.61 - 0.86)	0.14	49	0.083
3	*BANP-ZNF469*	rs9938149	3	Whites	C vs. A	1,030	6,757	**2.6×10^{-4}**	0.76 (0.66 - 0.88)	0.18	42	0.066
4	*COL5A1*	rs7044529	4	Whites	C vs. T	1335	7275	**9.9×10^{-4}**	0.81 (0.71 - 0.92)	0.41	12	0.12
5	*HGF*	rs3735520	5	Whites	T vs. C	1,101	4,356	**3.6×10^{-3}**	1.32 (1.10 - 1.60)	0.014	66	0.79
6	*LOX*	rs2956540	3	Whites	G vs. C	692	6,598	0.60	0.89 (0.57 - 1.38)	2.9×10^{-4}	91	6.0×10^{-5}
**Based on candidate gene studies**												
1	*COL4A4*	rs2229813	3	Whites	G vs. A	262	336	**1.3×10^{-12}**	2.38 (1.87 - 3.03)	0.68	0	0.39
		rs2229813	2	Chinese and Arabic	G vs. A	209	252	**0.047**	0.74 (0.56 - 1.00)	0.27	16	n.a.
		rs2228557	3	Whites	T vs. C	261	335	**4.5×10^{-7}**	0.54 (0.42 - 0.68)	0.59	0	0.31
		rs2229814	2	Whites	T vs. C	218	258	0.59	1.07 (0.83 - 1.39)	0.99	0	n.a.
		rs2228555	2	Whites	G vs. A	217	257	0.67	1.06 (0.82 - 1.38)	1.00	0	n.a.
2	*FNDC3B*	rs4894535	2	Chinese and Arabic	T vs. C	307	477	0.078	1.25 (0.98 - 1.60)	0.48	0	n.a.
3	*COL4A3*	rs55703767	3	Whites	T vs. G	262	334	0.11	0.19 (0.03 - 1.44)	3.0×10^{-14}	96	3.4×10^{-15}
		rs28381984	2	Whites	T vs. C	217	257	0.25	0.86 (0.66 - 1.11)	0.972	0	n.a.
		rs34019152	2	Whites	A vs. G	217	257	0.63	0.86 (0.47 - 1.58)	0.945	0	n.a.
		rs13424243	2	Whites	C vs. G	217	257	0.82	1.08 (0.57 - 2.04)	0.928	0	n.a.
		rs10178458	2	Whites	T vs. C	216	257	0.89	1.02 (0.73 - 1.44)	0.972	0	n.a.
		rs11677877	2	Whites	G vs. A	218	256	0.93	1.02 (0.62 - 1.69)	0.971	0	n.a.
		rs6436669	2	Whites	G vs. A	217	258	0.94	0.99 (0.70 - 1.40)	0.986	0	n.a.
4	*COL5A1*	rs7044529	3	Whites	C vs. T	684	4,514	0.11	0.88 (0.74 - 1.03)	0.678	0	0.52
		rs7044529	2	Chinese and Arabic	C vs. T	317	491	0.95	1.01 (0.73 - 1.40)	0.590	0	n.a.
5	*VSX1*	rs12480307	2	Chinese and Indian	G vs. A	214	209	0.29	2.06 (0.54 - 7.92)	0.163	49	n.a.
		rs6138482	2	Whites	A vs. G	155	149	0.59	1.10 (0.77 - 1.57)	0.411	0	n.a.
		rs6138482	3	Chinese, Korean, and Indian	A vs. G	459	406	0.98	1.01 (0.70 - 1.45)	0.068	64	0.026
6	*FOXO1*	rs2721051	2	Chinese and Arabic	C vs. T	314	489	0.31	0.79 (0.50 - 1.25)	0.377	0	n.a.
7	*BANP-ZNF469*	rs9938149	2	Chinese and Arabic	C vs. A	316	491	0.32	0.86 (0.65 - 1.15)	0.893	0	n.a.
8	*IL1B*	rs16944	3	Chinese, Korean, and Japanese	T vs. C	366	590	0.43	0.86 (0.60 - 1.25)	0.043	70	0.66
9	*MPDZ-NFIB*	rs1324183	2	Chinese and Arabic	C vs. A	317	491	0.63	0.87 (0.49 - 1.54)	0.020	82	n.a.

* A random-effects model was used.
† Asian ancestries included 2 or more ethnic groups from Arabic, Chinese, Korean, Japanese, or Indian populations.
CI=confidence interval; OR=odds ration; SNP = single nucleotide polymorphism; n.a. = not applicable; No. = number.

Supplementary Table 3. Quality assessment of case-control study using Newcastle - Ottawa scale (NOS)*

No.	First author (year)	Stars awarded	Selection				Comparability		Exposure		
			(1)	(2)	(3)	(4)	(1a)	(1b)	(1)	(2)	(3)
1	Abu-Amero, K. K., et al. (2015)	6	*	*	-	*	*	-	*	*	n.a.
2	Dudakova, L., et al. (2015)	5	*	*	-	*	*	-	*	-	n.a.
3	Hao, X. D., et al. (2015)	8	*	*	*	*	*	*	*	*	n.a.
4	Hasanian-Langroudi, F. (2015)	5	*	-	-	*	*	*	*	-	n.a.
5	Saravani, R. (2015)	6	*	-	-	*	*	*	*	*	n.a.
6	Kokolakis, N. S. (2014)	7	*	-	*	*	*	*	*	*	n.a.
7	Karolak, J. A., et al. (2014)	6	*	*	-	*	*	-	*	*	n.a.
8	Sahebjada, S. (2014)	6	*	-	*	*	*	-	*	*	n.a.
9	Palamar, M. (2014)	6	*	-	-	*	*	*	*	*	n.a.
10	Bae, H. A. (2013)	8	*	*	*	*	*	*	*	*	n.a.
11	Li, X. (2013)	6	*	*	*	-	*	-	*	*	n.a.
12	Sahebjada, S. (2013)	6	*	-	*	*	*	-	*	*	n.a.
13	Mikami, T. (2013)	6	*	-	-	*	*	*	*	*	n.a.
14	Verma, A. (2013)	5	*	-	-	*	*	-	*	*	n.a.
15	Lu, Y. (2013)†	7	*	*	*	*	*	-	*	*	n.a.
16	Wang, Y. (2013)	6	*	-	-	*	*	*	*	*	n.a.
17	Bykhovskaya, Y. (2012)†	6	*	*	*	*	*	-	*	-	n.a.
18	Li, X. (2012)†	6	*	*	*	*	*	-	*	*	n.a.
19	Burdon, K. P. (2011)†	6	*	*	*	*	*	-	*	*	n.a.
20	Stabuc-Silih, M. (2010)	5	*	-	-	*	*	-	*	*	n.a.
21	Stabuc-Silih, M. (2010)	5	*	-	-	*	*	-	*	*	n.a.
22	Stabuc-Silih, M. (2009)	5	*	-	-	*	*	-	*	*	n.a.
23	Kim, S. H. (2008)	8	*	*	*	*	*	*	*	*	n.a.
24	Mok, J. W. (2008)	7	*	*	*	*	*	-	*	*	n.a.

*Newcastle - Ottawa Quality assessment scale was detailed in Supplementary Appendix 1.

† Genome-wide association study

"-" means no star was awarded. n.a. = not applicable.

Supplementary Table 4. Sensitivity analysis of allelic associations by excluding cohorts deviated from Hardy-Weinberg Equilibrium

No.	Gene/locus	SNP	No. of cohorts	Ethnicity	Associated allele vs. Reference allele	Pooled sample size		Outcome*		Heterogeneity		Egger's test (P)
						Case	Control	P	OR (95% CI)	P (Q)	I^2 (%)	
**Based on both GWAS and replication studies**												
1	*FOXO1*	rs2721051	4	Multiple ancestries†	C vs. T	1,139	7,058	**4.4×10^{-11}**	0.64 (0.56 - 0.73)	0.490	0	0.83
**Based on candidate gene studies**												
1	*FOXO1*	rs2721051	2	Multiple ancestries†	C vs. T	265	974	**7.5×10^{-3}**	0.68 (0.51 - 0.90)	0.523	0	n.a.
2	*COL4A3*	rs10178458	2	Multiple ancestries†	T vs. C	209	201	0.87	0.97 (0.65 - 1.44)	0.745	0	n.a.
		rs55703767	3	Multiple ancestries†	T vs. G	256	279	0.43	0.46 (0.07 - 3.08)	3.1×10^{-11}	96	0.45
		rs55703767	2	Whites	T vs. G	158	177	0.46	0.32 (0.016 - 6.54)	3.1×10^{-11}	98	n.a.
3	*COL4A4*	rs2228555	2	Multiple ancestries†	G vs. A	225	250	0.86	1.02 (0.79 - 1.33)	0.807	0	n.a.
		rs2229813	4	Multiple ancestries†	G vs. A	367	431	0.46	1.27 (0.68 - 2.40)	1.3×10^{-6}	89	0.42
		rs2229813	2	Whites	G vs. A	158	179	**3.5×10^{-7}**	2.30 (1.67 - 3.16)	0.42	0	n.a.
		rs2229814	2	Multiple ancestries†	T vs. C	211	201	0.95	1.01 (0.77 - 1.33)	0.634	0	n.a.
4	*VSX1*	rs12480307	2	Multiple ancestries†	G vs. A	159	158	0.23	1.27 (0.86 - 1.88)	0.625	0	n.a.

* A random-effects model was used.

† Multiple ancestries included 2 or more ethnic groups from Whites and Asian (i.e., Arabic, Chinese, Korean, Japanese, or Indian).

CI=confidence interval; OR=odds ration; SNP = single nucleotide polymorphism; n.a. = not applicable; No. = number.

Supplementary Table 5. Search strategies for MEDLINE and Embase databases on the Ovid platform

No.	Search terms
1	polymorphism*.mp.
2	nucleotide*.mp.
3	variant*.mp.
4	genom*.mp.
5	mutation*.mp.
6	(locus or loci).mp.
7	DNA.mp.
8	intron.mp.
9	(exon or exome).mp.
10	allel*.mp.
11	haplotype*.mp.
12	genotyp*.mp.
13	genetic*.mp.
14	1 or 2 or 3 or 4 or 5 or 6 or 7 or 8 or 9 or 10 or 11 or 12 or 13
15	keratoconus.mp. or exp Keratoconus/
16	14 and 15

No.=number

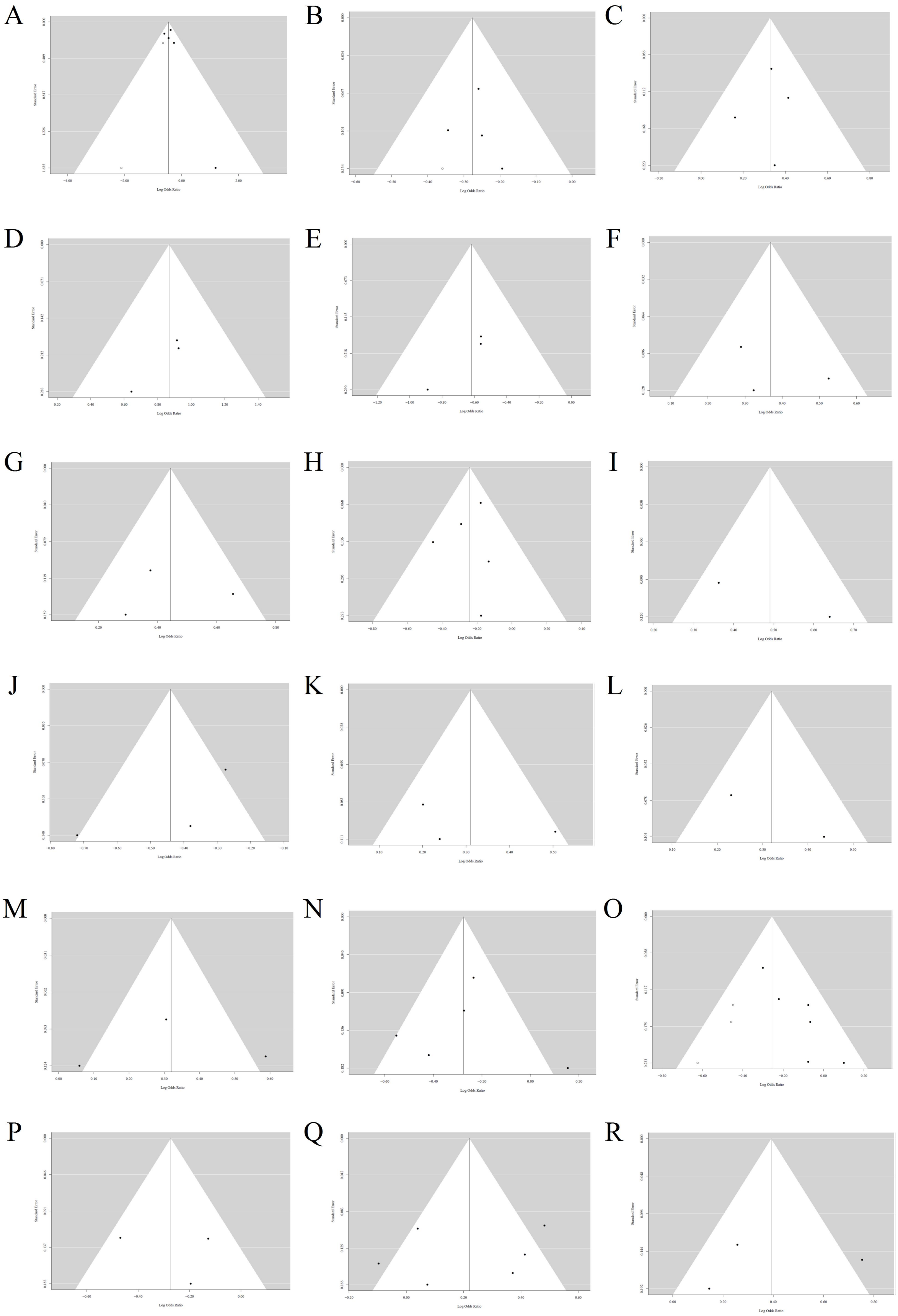
A
B
C
D
E
F
G
H
I
J
K
L
M
N
O
P
Q
R

Supplementary Figure 1. Funnel plots of significant genetic associations for keratoconus

A: *FOXO1* rs2721051 (P_{meta}=**5.6×10^{-11}**, P_{Egger}=0.35**);** B: *RXRA-COL5A1* rs1536482 (P_{meta}=**2.5×10^{-9}**, P_{Egger}=0.89); C: *FNDC3B* rs4894535 (P_{meta}=**1.4×10^{-8}**, P_{Egger}=0.76); D: *COL4A4* rs2229813 (P_{meta}=**1.3×10^{-12}**, P_{Egger}=0.39); E: *COL4A4* rs2228557 (P_{meta}=**4.5×10^{-7}**, P_{Egger}=0.31); F: *IMMP2L* rs757219 (P_{meta}=**6.1×10^{-7}**, P_{Egger}=0.61); G: *IMMP2L* rs214884 (P_{meta}=**2.3×10^{-5}**, P_{Egger}=0.89); H: *BANP-ZNF469* rs9938149 (P_{meta}=**1.3×10^{-5}**, P_{Egger}=0.77); I: *KCND3* rs4839200 (P_{meta}=**3.9×10^{-4}**, P_{Egger}=n.a.); J: *RAB3GAP1* rs4954218 (P_{meta}=**8.2×10^{-4}**, P_{Egger}=0.19); K: *UBXD2* rs6430585 (P_{meta}=**1.1×10^{-3}**, P_{Egger}=0.62); L*: 13q33.3* rs1328089 (P_{meta}=**1.7×10^{-3}**, P_{Egger}=n.a.); M: *13q33.3* rs1328083 (P_{meta}=**3.0×10^{-2}**, P_{Egger}=0.88); N: *MPDZ-NFIB* rs1324183 (P_{meta}=**5.5×10^{-3}**, P_{Egger}=0.75); O: *COL5A1* rs7044529 (P_{meta}=**7.0×10^{-3}**, P_{Egger}=0.051); P: *LOX* rs10519694 (P_{meta}=**0.018**, P_{Egger}=0.74); Q: *HGF* rs3735520 (P_{meta}=**0.027**, P_{Egger}=0.60); R: *19p12* rs8111998 (P_{meta}=**0.035**, P_{Egger}=0.74); n.a.=not applicable

Supplementary Appendix 1

NEWCASTLE - OTTAWA QUALITY ASSESSMENT SCALE

CASE CONTROL STUDIES

(accessed via http://www.ohri.ca/programs/clinical_epidemiology/oxford.asp)

Note: A study can be awarded a maximum of one star for each numbered item within the Selection and Exposure categories. A maximum of two stars can be given for Comparability.

Selection

1) Is the case definition adequate?
   - a) yes, with independent validation
   - b) yes, e.g. record linkage or based on self reports
   - c) no description

2) Representativeness of the cases
   - a) consecutive or obviously representative series of cases
   - b) potential for selection biases or not stated

3) Selection of Controls
   - a) community controls
   - b) hospital controls
   - c) no description

4) Definition of Controls
   - a) no history of disease (endpoint)
   - b) no description of source

Comparability

1) Comparability of cases and controls on the basis of the design or analysis
   - a) study controls for ________________ (Select the most important factor: ethnicity)
   - b) study controls for any additional factor (This criteria could be modified to indicate specific control for a second important factor.)

Exposure

1) Ascertainment of exposure
   - a) secure record (e.g. surgical records)
   - b) structured interview where blind to case/control status
   - c) interview not blinded to case/control status
   - d) written self report or medical record only
   - e) no description

2) Same method of ascertainment for cases and controls
   - a) yes
   - b) no

3) Non-Response rate (*This item was not applicable for this study.)

a) same rate for both groups

b) non respondents described

c) rate different and no designation
